# The Conquest of Lead Poisoning: A Pyrrhic Victory

**DOI:** 10.1289/ehp.10871

**Published:** 2007-10

**Authors:** Bruce P. Lanphear

**Affiliations:** Cincinnati Children’s Environmental Health Center, Cincinnati Children’s Hospital Medical Center, University of Cincinnat, Cincinnati, Ohio, E-mail: bruce.lanphear@cchmc.org

The dramatic decline in childhood lead poisoning in the United States has often been declared a public health victory. The history of lead poisoning has all the essential elements of a successful campaign: A long, embittered battle was waged by a small cadre of intrepid parents, scientists, policy makers, and physicians against government inertia and industry opposition, and they won. The death toll from overt lead poisoning was staunched. In the 1960s, thousands of children with lead encephalopathy were hospitalized each year in the United States; about one in four died (Christian et al. 1964; Greengard et al. 1965). In contrast, only one child died from overt lead poisoning in the past decade [Centers for Disease Control and Prevention (CDC) 2006]. Over the past three decades, blood lead levels of children and adults have plummeted as a result of bans on lead in gasoline, paint, and solder used in canned foods (CDC 2005; Pirkle et al. 1998).

But recent evidence and events—as well as the perpetually tardy and too often insufficient regulatory efforts—all raise serious doubts about whether the decline in lead poisoning should be declared a victory (Lanphear et al. 2003).

Lead toxicity remains a global problem. Despite some success in the worldwide ban of leaded gasoline, widespread lead exposure from industrial emissions and lead-contaminated paint and consumer products remains common among children in many parts of the world (Tong et al. 2000). Levels of lead exposure previously thought to be safe or inconsequential for children have consistently been shown to be risk factors for reading problems, intellectual delays, school failure, attention deficit–hyperactivity disorder, and antisocial behaviors (Bellinger 2004; Braun et al. 2006; Burns et al. 1999; Dietrich et al. 2001; Needleman et al. 1990, 1996). No evidence shows that there is a threshold for the adverse effects of lead exposure; indeed, compelling evidence indicates that lead-associated decrements in intellectual function are proportionately greater at a blood lead level < 10 μg/dL (Kordas et al. 2006; Lanphear et al. 2005a; Schwartz 1994; Tellez-Rojo et al. 2006).

Low-level lead toxicity is not confined to childhood. Considerable evidence implicates lead exposure commonly found in the U.S. population as a risk factor for disability and disease in adults, including cognitive decline and cardiovascular disease (Menke et al. 2006; Schwartz et al. 2005; Weisskopf et al. 2004). This evidence—which first began to surface in the scientific literature during the 1970s—suggests that the consequences of lead exposure for children born during the latter half of the 20th century will persist into the first half of the 21st century.

In light of the prophetic, but largely ignored, warnings about the hazards of using lead in paint, gasoline, and consumer products (Markowitz et al. 2000; Rabin 1989; Rosner et al. 1985), it is presumptuous to declare the decline in childhood lead poisoning a public health victory. If it is a victory, it most certainly is a Pyrrhic one.

It is easy to blame the chief culprits of the epidemic—the paint and pigment industry, the petroleum industry, and a few industry-funded scientists (Markowitz et al. 2000; Rabin 1989; Rosner et al. 1985). But the reasons for the delays in regulation are more complex than the nefarious actions of a few profiteers. For too long, we chose to deny the burgeoning evidence about lead toxicity. Swayed by industry’s expertly packaged arguments, public health officials and pediatricians found it convenient to blame the consequences of lead toxicity on poverty, poor parenting, or pica. Meanwhile, epidemiologists fretted about unmeasured confounders and the limitations of observational studies. In our quest for scientific certainty, we inadvertently delayed the promulgation of regulations at the expense of public health.

Despite conclusive evidence that regulations led to the dramatic decline in lead poisoning over the past three decades, we continue to rely on obsolete and insufficient secondary prevention strategies to protect contemporary children from lead hazards (Lanphear 1998; Lanphear et al. 2003). The key to primary prevention is to eliminate environmental lead exposure. This will, first and foremost, require a declaration of the full scope of the problem; society cannot respond to a threat until it first acknowledges it. It will require the promulgation of regulations to further reduce environmental lead exposure; the global phaseout of leaded gasoline; screening of high-risk, older housing units to identify lead hazards before a child is exposed—before occupancy, after renovation or abatement; control of industrial emissions; and stricter regulations and enforcement on the allowable levels of lead in toys, jewelry, and other consumer products. Finally, it will require a worldwide ban on all nonessential uses of lead that pose a threat to human or ecologic health.

Over the past 100 years, since the recognition of lead poisoning as a distinct threat to children, several other environmental toxicants have been identified that adversely affect children, including polychlorinated biphenyls, tobacco, mercury, manganese, and arsenic (Grandjean et al. 1997; Rosado et al. 2007; Schantz et al. 2003; Wasserman et al. 2006; Weitzman et al. 2002). There is emerging evidence that other chemicals—many of which are readily found in the blood and tissues of pregnant women and children but have not been sufficiently tested for toxicity—may be causing serious adverse health effects (Eskenazi et al. 2007; Needham et al. 2005; Rauh et al. 2006; Swan et al. 2005). Even if we were victorious in the battle against lead poisoning, it would be a victory diminished by our failure to learn from the epidemic and take steps to dramatically reduce exposures to other confirmed and suspected environmental toxicants as well as chemicals of uncertain toxicity (Lanphear et al. 2005b).

## Figures and Tables

**Figure:**
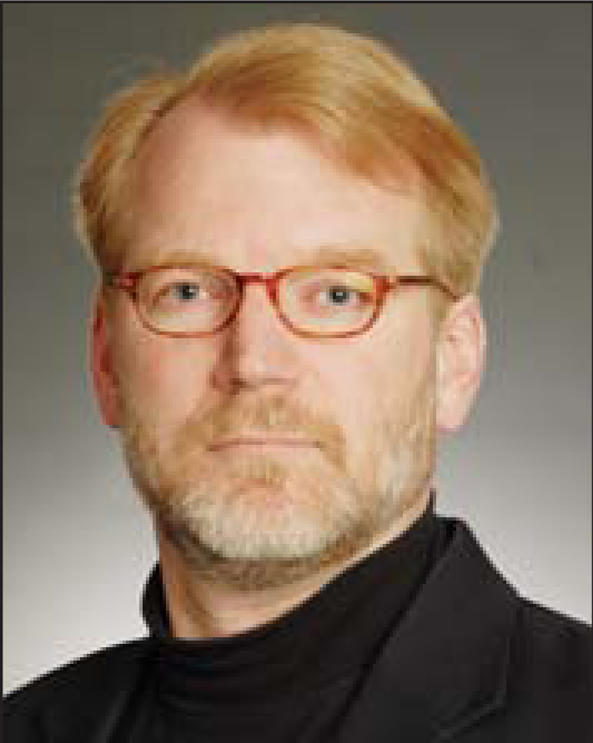
Bruce P. Lanphear
